# *TNF-α* -308 G > A (rs1800629) Polymorphism is Associated with Celiac Disease: A Meta-analysis of 11 Case-Control Studies

**DOI:** 10.1038/srep32677

**Published:** 2016-09-06

**Authors:** Saif Khan, Raju K. Mandal, Arshad Jawed, Sajad A. Dar, Mohd Wahid, Aditya K. Panda, Mohammed Y. Areeshi, Md. Ekhlaque Ahmed Khan, Shafiul Haque

**Affiliations:** 1 Department of Clinical Nutrition, College of Applied Medical Sciences, University of Ha’il, Ha’il-2440, Saudi Arabia; 2 Research and Scientific Studies Unit, College of Nursing & Allied Health Sciences, Jazan University, Jazan-45142, Saudi Arabia; 3 The University College of Medical Sciences & GTB Hospital (University of Delhi), Delhi-110095, India; 4 Department of Biosciences, Faculty of Natural Sciences, Jamia Millia Islamia (A Central University), New Delhi-110025, India; 5 Centre for Life Sciences, Central University of Jharkhand, Brambe, Ranchi-835205, Jharkhand, India

## Abstract

Celiac disease (CD) remains one of the most significant autoimmune diseases worldwide. The pathogenesis of CD is not clearly understood and is probably attributed to genomic variations and host genetic make-up. Case-control and cohort studies of the association between the *TNF-α* -308 G > A (rs1800629) polymorphism and CD susceptibility have yielded inconsistent results. In this study, PubMed, EMBASE, and Google Scholar web-databases were searched for pertinent reports showing association of *TNF-α* -308 G > A gene with CD risk. A total of eleven reports involving 1774 controls and 1147 CD cases were included. Significant associations in four genetic models, *viz.* variant allele (A vs. G: p = 0.001; OR = 2.051, 95% CI = 1.452–2.895), variant homozygous (AA vs. GG: p = 0.001; OR = 6.626, 95% CI = 3.569–12.300), recessive (AA vs. GG + AG: p = 0.001; OR = 4.766, 95% CI = 3.177–7.152) and dominant (AA + AG vs. GG: p = 0.008; OR = 1.910, 95% CI = 1.181–3.088) were found in comparison with wild type homozygous GG genotype. However, heterozygous genetic model did not show any association. Sensitivity analysis revealed stable and statistically robust results. Our results suggest that *TNF-α* -308 G > A gene polymorphism significantly contributes to CD susceptibility.

Celiac disease (CD; also known as celiac sprue, gluten-sensitive enteropathy, and nontropical sprue) is a chronic inflammatory autoimmune disorder characterized by abnormalities in the small intestine caused by permanent intolerance to dietary gluten or related proteins from wheat and rye^1^. Although few CD patients may suffer primarily from gastrointestinal symptoms, CD may be related with extra-intestinal disorders^2^. Genetic, immunological and environmental factors have been attributed for the disease. Till date, the etiology of CD has not yet been fully elucidated. However, CD has shown a strong genetic relation with HLA (human leucocyte antigen) class II gene, contributing no more than 40% to the disease risk, which shows involvement of genetic components in the development of CD^3^,^4^. Recently published genome-wide association study (GWAS) has identified several non-HLA genes as new susceptibility factors for CD. So far, 39 loci with 57 independent association signals have been defined and many of these loci harbour genes associated with immunity^5^. This suggests that genetic susceptibility to CD is also conferred by some other non HLA genes. Earlier, studies have revealed that genes encoding for the pro-inflammatory cytokines could predispose to immunological mediated lesion of CD^6^. Cytokines are important mediators of immunity and their responses due to imbalance or deficiency in the cytokine network may largely determine autoimmune disease susceptibility and severity.

Tumor necrosis factor-alpha (TNF-α) is a potentpro-inflammatory and immunoregulatory cytokine mapped on chromosome 6 (6p21.31) spanning about 3 kb and contains 4 exons. TNF-α stimulates many other cytokines and mediates the cytokine cascade that causes inflammation^7^. *TNF-α* gene has close linkage between HLA class I and class II^8^, and tightly regulated at the level of transcription^9^. Previous studies reported that sequence variation in the regulatory region of *TNF-α* gene has been correlated with various autoimmune diseases^10^. Several biallelic single nucleotide polymorphisms (SNPs) have been noted in the *TNF-α* gene^11^. Among them one G (guanine) >A (adenine) polymorphism is located upstream of the gene at -308 and is known to influence TNF-α levels. In comparison with the *TNF-α* -308G allele, A allele has higher transcriptional activity and often connected to autoimmune diseases^12^,^13^. The location of the gene within the major histocompatibility complex and the putative role of -308 G > A polymorphism on the promoter activity of *TNF-α* gene has raised the possibility that this polymorphism may influence immunologic homeostasis and contribute to the pathogenesis of CD.

Keeping aforesaid information in view, to date a number of case control and cohort studies have been performed to investigate the association of *TNF-α* -308 G > A gene polymorphism and CD susceptibility^14^,^15^,^16^,^17^,^18^,^19^,^20^,^21^,^22^,^23^,^24^. However, the results are inconsistent and controversial because of small sample size of individual study and possible selective bias. In order to overcome the limitations of single studies, we conducted this meta-analysis by pooling previous single studies to increase the statistical power and to derive more precise and comprehensive relationship between *TNF-α* -308 G > A gene polymorphism and CD susceptibility. The schematic representation of the entire pooled study is presented as Graphical Abstract (Fig. 1).

## Results

### Characteristics of the selected published studies

**Figure 2:**
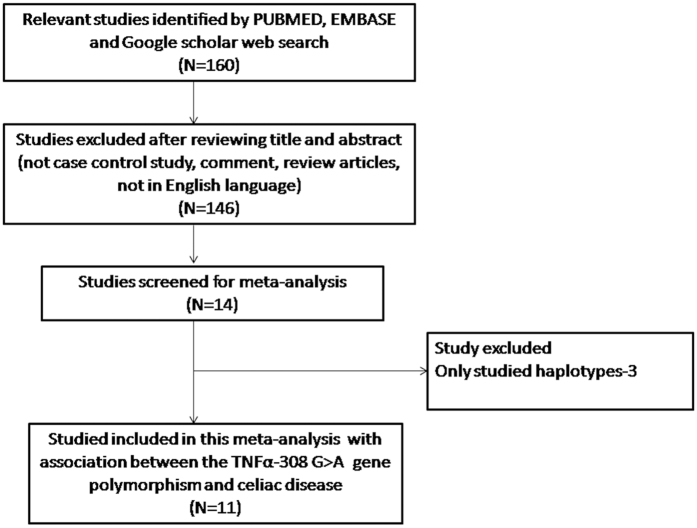
PRISMA flow-diagram showing the selection process (inclusion/exclusion) of the pertinent studies of *TNF-α* -308 G > A (rs1800629) polymorphism and CD risk.

The number of hits obtained by doing a literature search via PubMed (Medline), EMBASE and Google Scholar search database were one hundred and sixty. All the retrieved hits (articles) were checked by reading their titles and abstracts, and the full texts for the possibly relevant publications. In addition, the articles were further scrutinized for their appropriateness for this meta-analysis (Fig. 2: PRISMA Flow-diagram). Likewise, the references listed in the retrieved publications were also screened for other possible apposite articles. In order to derive a precise conclusion from this pooled analysis, very stringent criteria were followed for searching and selecting the pertinent publications, for e.g., only case-control or cohort design studies with frequencies of all the three genotypes were included. After thorough analysis and following the stern criteria of selection (inclusion or exclusion), eleven originally published studies representing the above mentioned possible association were found eligible and included in this study (Table 1). A PRISMA flow-diagram showing the selection process (inclusion/exclusion) of the studies for this meta-analysis is given in Fig. 2. Information regarding distribution of genotypes, HWE p-values in the controls, and susceptibility to CD is provided in Table 2. All the eleven studies were assessed for the quality according to the Newcastle-Ottawa Scale and most studies (80%) scored 5 stars or more, suggesting a moderate to good quality (Table 3).

### Evaluation of publication bias

**Figure 3:**
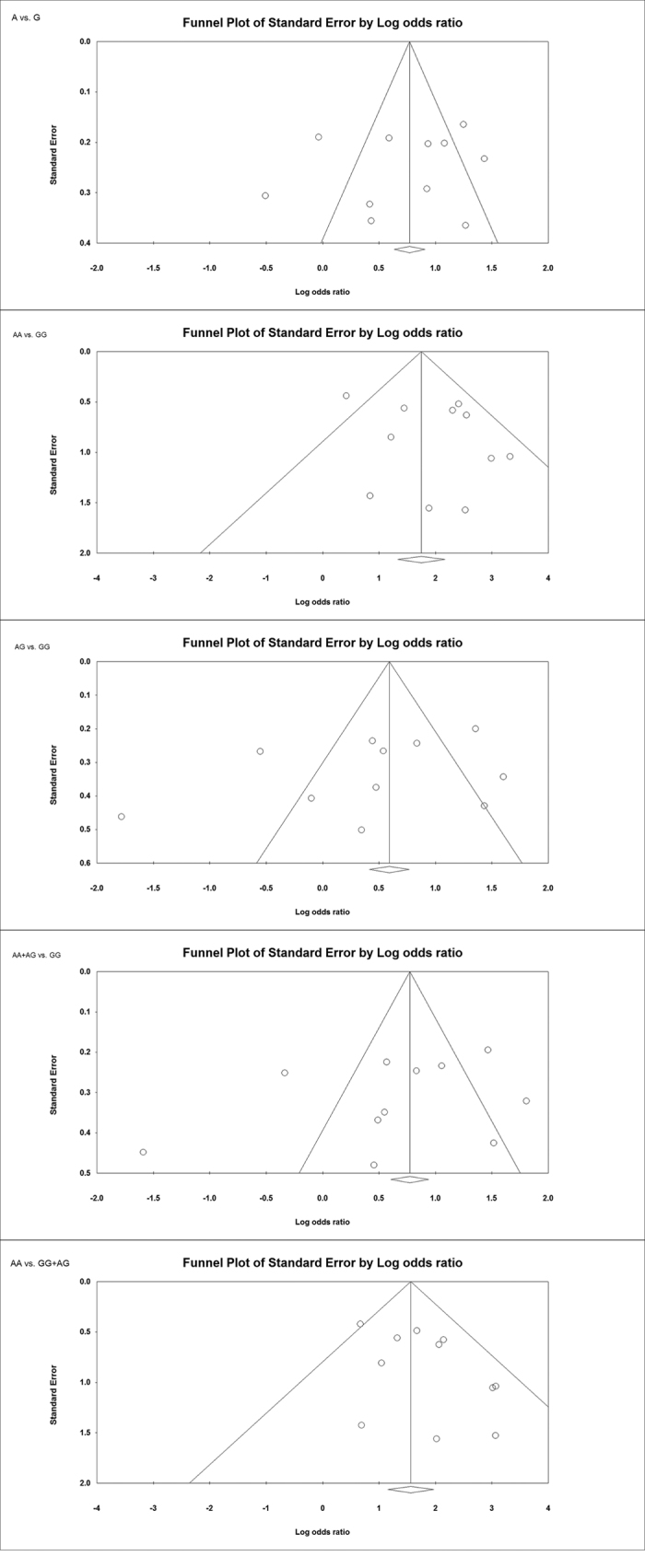
Assessment of publication bias shown for all the genetic models (allele: A vs. G, homozygous: AA vs. GG, heterozygous: AG vs. GG, dominant: AA + AG vs. GG, recessive: AA vs. GG + AG) with Funnel plots in studies assaying odds of CD associated with *TNF-α* -308 G > A polymorphism.

Funnel plot and Egger’s test were employed to test publication bias among the selected studies (Table 4; Fig. 3). The Funnel plots remained symmetric and Egger’s linear regression test also indicated no evidence of publication bias among the studies testing -308 G > A polymorphism of *TNF-α* gene and CD susceptibility (Fig. 3).

### Test of heterogeneity

Q-test and I^2^ statistics were used to evaluate the inter- and intra-study variations, and based upon the significance value different models were selected for the present meta-analysis (Table 4). Heterogeneity was observed in three genetic models, i.e., variant allele (A vs. G) heterozygous (AG vs. GG) and recessive (AA + AG vs. GG), hence random effects model was applied.

### Meta-analysis of *TNF-α* -308 G > A polymorphism and CD susceptibility

**Figure 4:**
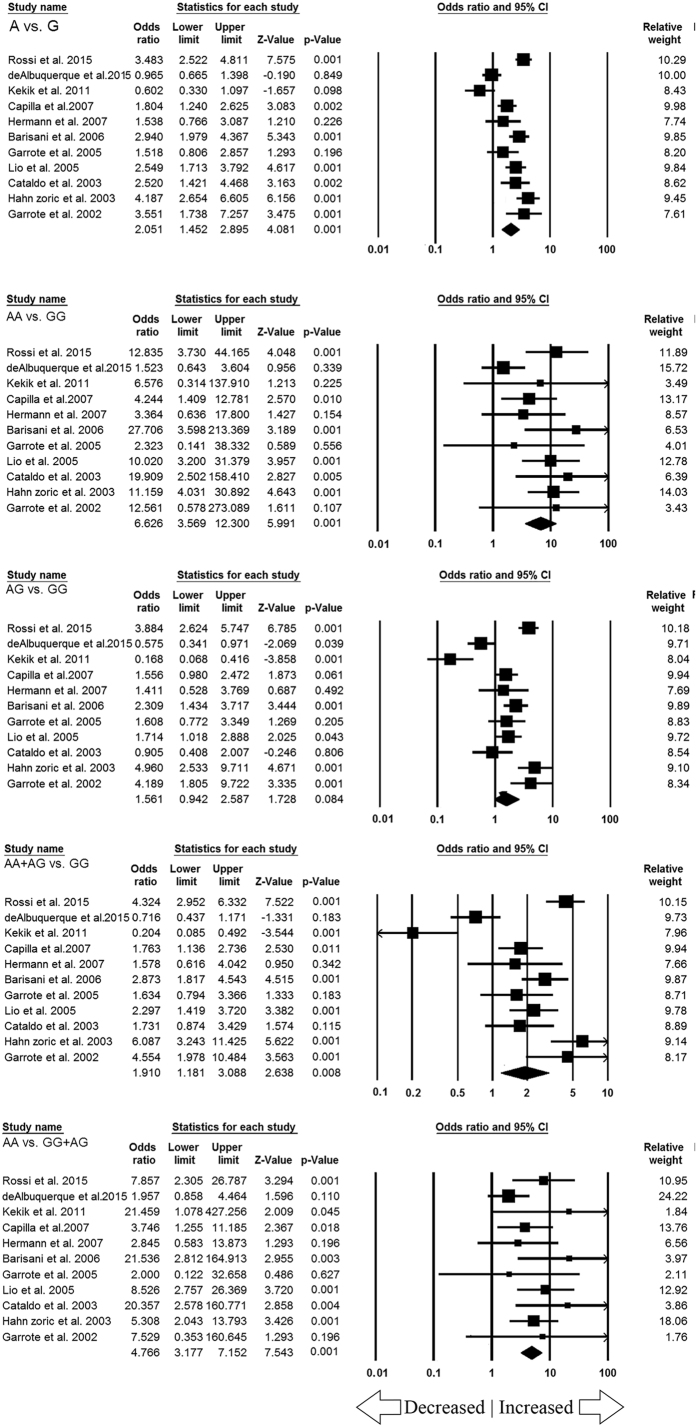
Forest plot for overall analysis (allele: A vs. G, homozygous: AA vs. GG, heterozygous: AG vs. GG, dominant: AA + AG vs. GG, recessive: AA vs. GG + AG) showing OR with 95% CI to evaluate the association of *TNF-α* -308 G > A (rs1800629) polymorphism and CD risk. Note: Black square represents the value of OR and the size of the square indicates the inverse proportion relative to its variance. Horizontal line is the 95% CI of OR.

After cautious appraisal, all the data from the selected eleven studies were pooled together that lead to 1774 controls and 1147 CD cases were included to evaluate the overall association between the *TNF-α* -308 G > A gene polymorphism and the risk of CD. Overall significant increased risk of CD susceptibility was found in variant allele (A vs. G: p = 0.001; OR = 2.051, 95% CI = 1.452–2.895), homozygous variant (AA vs. GG: p = 0.001; OR = 6.626, 95% CI = 3.569–12.300), recessive (AA vs. GG + AG: p = 0.001; OR = 4.766, 95% CI = 3.177–7.152) and dominant (AA + AG vs. GG: p = 0.008; OR = 1.910, 95% CI = 1.181–3.088) genetic models in comparison with wild type GG genotype. However heterozygous (AG vs. GG: p = 0.084; OR = 1.561, 95% CI = 0.942–2.587) genetic model did not show any association. The Forest plots for all the genetic models are shown in Fig. 4.

### Sensitivity analysis

**Figure 5:**
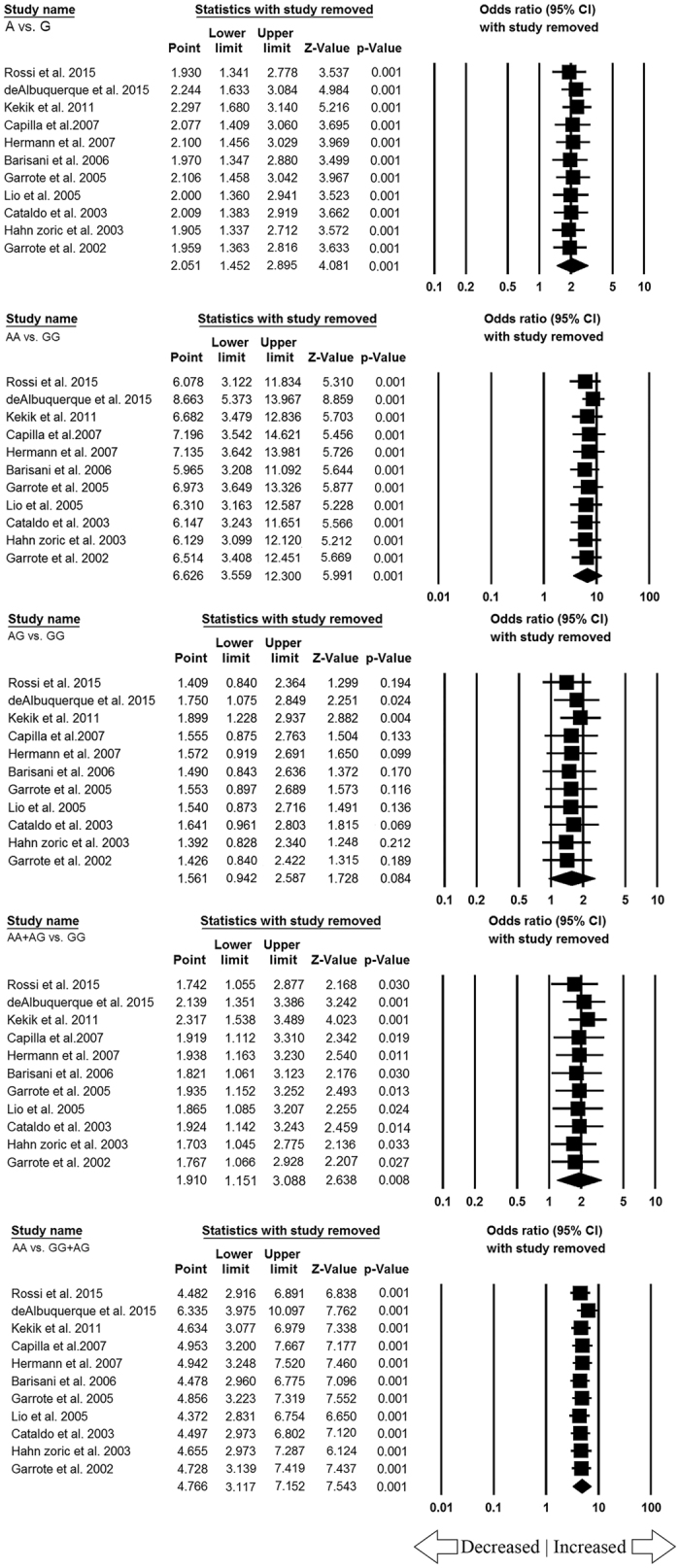
Sensitivity analysis showing all the genetic models (allele: A vs. G, homozygous: AA vs. GG, heterozygous: AG vs. GG, dominant: AA + AG vs. GG, recessive: AA vs. GG + AG).

One way sensitivity analysis was performed to appraise the effect of each individual study on the pooled ORs by serially deleting one single study each time. When omitted each study in the current meta-analysis, the pooled ORs were always remains the same and no other single study influenced the pooled ORs. This suggests that the results of *TNF-α* -308 G > A gene polymorphism and CD susceptibility were stable and statistically robust (Fig. 5).

## Discussion

Celiac disease (CD) is one of the best understood autoimmune disorders and genetic factors are considered to be strong determinants of this disease^25^, which encourage researchers to search for the responsible genes for CD susceptibility. TNF-α is pleiotropic cytokine mediates broad range of proinflammatory and inflammatory responses and alter levels of TNF-α among different individuals can be a major risk for differences in the susceptibility and severity of CD. Most of the evidences suggest that the endogenous production of TNF-α may be influenced by TNF-α promoter polymorphisms, thereby affecting messenger RNA (mRNA) and protein expression levels^26^. In view of facts about the significant role of *TNF-α* gene in modulation of acute inflammation and host innate immunity, various studies have been conducted to analyze the relationship between *TNF-α* -308 G > A gene polymorphism and the development of CD, but the results from different published studies were contradictory and inconclusive^14^,^15^,^16^,^17^,^18^,^19^,^20^,^21^,^22^,^23^,^24^. In order to improve the statistical power, we performed the present meta-analysis to assess the said association of *TNF-α* -308 G > A polymorphism and CD risk from eleven case-control studies, as combining of the data from different studies has an advantage of reducing the random error^27^. In the current meta-analysis, we separately extracted different genotype frequencies of the *TNF-α* -308 G > A polymorphism in CD and estimated the pooled ORs and their corresponding 95% CI in all the genetic models. Our meta-analysis showed that ORs of the four genetic models were on the right side of the vertical line and the corresponding 95% CI of the pooled ORs were also on the right side of the vertical line with a significant p-value. This suggest that *TNF-α* -308 G > A gene polymorphism might contribute to functional role in the pathogenesis of CD. Earlier studies have suggested the role of *TNF-α* gene in CD development, and it has been observed that *TNF-α* -308 A allele directly affects the transcriptional activity that leads to higher production of TNF-α in *in-vitro* experiment^28^ and higher levels of TNF-α transcription has been reported to facilitate the inflammatory response to gluten^29^. Genetic variants in the *TNF-α* gene might increase the production of TNF-α for prolonged duration and cause uncontrolled or severe inflammation and influences the increased risk of CD. Also, up-regulated TNF-α expression has been observed in epithelial cells and intraepithelial lymphocytes in the mucosa of CD patients^30^. TNF-α also triggers a proteolytic cascade mediated by matrix metalloproteinases (MMPs) secretion from intestinal myofibroblasts and results in intestinal architectural alteration^31^. Therefore, blockade of this cytokine may prevent the activation of proteolytic MMPs and ultimately resumes the intestinal haemostasis. Earlier studies have reported the use of monoclonal antibodies against TNF-α (infliximab), beneficial for patients with severe refractory CD and uncontrolled sprue^32^,^33^. However, larger scale clinical trial studies are necessary to demonstrate the clinical utility of *TNF-α* -308 G > A polymorphism for the diagnosis and the treatment of CD.

The genetics involved in the development of CD is very complex, with evidence for the participation of multiple intrinsic (genetic) and extrinsic (environmental) factors^34^, hence, a single genetic variant is generally inadequate and failed to interpret the risk of this disease. Notwithstanding, the significant findings achieved from this study, we still have to acknowledge some of the limitations of this meta-analysis. Firstly, we found heterogeneity in this pooled study, which might be attributed to one or more of the following reasons, the ethnic origin of the patients, recruiting control samples and quality of studies. Second, language bias may exist because reports published in the English language were only considered for this meta-analysis. Third, we only included published articles available in PubMed, EMBASE, and Google Scholar web databases, pertinent articles published in other databases, print only journals, and unpublished (i.e., studies reported other than research articles in journals, for e.g., doctoral/academic thesis etc.) studies may have been missed. Fourth, meta-analysis remains a retrospective research, which is subject to the methodological deficiencies or selection bias of the included studies and may possibly influence or deviate the reliability of our study results^35^.

Nevertheless, our current meta-analysis has some advantages, which could be summarizes as follows. First, we tried our best to find as many published studies by means of various searching approaches. Second, publication bias was not detected by funnel plot and Egger’slinear regression test, thus the all results were statistically robust. Third, sensitivity analysis also demonstrated that the results were not influenced by any single study. Fourth, we used strict data extraction and analysis to make satisfactory and reliable conclusion.

In conclusion, we can say that meta-analysis is an extremely valuable and powerful tool for numerical data-analysis that takes in to account both statistically significant and non-significant data from the individual studies and results into a cumulative precise conclusion. To the best of our knowledge, this is the very first meta-analysis showing association of *TNF-α* -308 G > A polymorphism with increased CD susceptibility. Hence, determining *TNF-α* -308A genotype is a probable determinant for the development of CD. Further, well designed large-scale studies with the considerations of gene-gene and gene-environment interactions are warranted to investigate this association. Future studies are encouraged to validate our current findings and to prove the clinical relevance of *TNF-α* -308 G > A polymorphism in the development of CD. Here, we analyzed the -308 G > A variant of *TNF-α* gene for the risk of CD without considering the interaction between several other SNPs. In future, we intend to explore other pertinent interactions to facilitate the discovery of CD development.

## Methods

### Identification of eligible studies

Pertinent studies were cautiously identified by multiple comprehensive search of PubMed (Medline), EMBASE and Google Scholar online web databases covering all research articles published with a combination of the following key words: ‘Tumor necrosis factor-alpha gene OR *TNF-α* gene OR *TNF* gene AND polymorphism OR mutation OR variant AND -308 rs1800629 AND Celiac OR Coeliac disease’ (last updated on May 2016). All the references present in the retrieved studies were also checked by hand search to identify the studies that possibly have not been included in these databases. All the published studies matching with the above mentioned eligibility criteria were selected and included in the present meta-analysis.

### Inclusion and exclusion criteria for the selection of studies

In order to minimize heterogeneity and facilitate the accuracy of the findings, studies included in the present meta-analysis had to follow the preset criteria: (i) must evaluated *TNF-α* -308 G > A polymorphism and CD risk, (ii) reported original data from the case-control and cohort study, (iii) enrolled pathologically confirmed CD cases and CD-free controls, (iv) presented useful genotype frequency in all the cases as well as in the controls, and (v) must be published in the English language. The leading reasons for the exclusion of the studies were, overlapping of the data, review articles, abstract only, and case-only studies. The studies of TNF polymorphism to predict survival and expression level considering it as an indicator for the response to therapy were also excluded.

### Data extraction

For all the selected publications, the methodological quality assessment and the data extraction were independently abstracted in duplicate by two independent investigators using a standard protocol. The data accuracy was determined by using a data-collection form according to the pre-set inclusion criteria as stated in the above section. The quality assessment of the case-control studies for this meta-analysis was done by two independent investigators (AJ & SAD) based on the pre-set eligibility criteria of the present study, and sequential exclusion of the inappropriate studies. In case of disagreements between the two investigators on any item regarding the data collected from the retrieved studies, the issue was openly discussed in detail and an agreement was reached following a discussion with the adjudicator (RKM). The abstracted characteristics from the selected studies included the name of first author, the year of publication, the country of origin, sources of study, the number of cases/controls, genotyping methods and frequencies and association with CD.

### Quality assessment of the selected studies

Methodological quality assessment of the included studies was done separately by two independent researchers using the Newcastle-Ottawa Scale (NOS) criteria^36^. The NOS criteria included three aspects: (1) subject selection: 0–4 points; (2) comparability of subject: 0–2 points; (3) clinical outcome: 0–3 points. Studies that were awarded 5 stars or more can be considered as of medium to high quality^37^.

### Statistical analysis

The pooled odds ratios (ORs) and their respective 95% confidence intervals (CIs) were calculated to evaluate the relation between *TNF-α* -308 G > A gene polymorphism and CD risk. Heterogeneity belief associated with the selected studies was investigated by the chi-square based Q-test and I^2^ test^38^. The heterogeneity was considered significant if the p-value was less than 0.1. While calculating the pooled ORs, fixed-effects models were used if the I^2^ value was less than 70%; otherwise, random-effects models were adopted^39^,^40^. Hardy-Weinberg equilibrium (HWE) is an application of the binomial theorem to population genetics and stating that the genetic variation in a population will remain constant from one generation to the next in the absence of disturbing factors. The assessment of departure from HWE is performed by chi-square test in the controls because a deviation from HWE in the cases might indicate a genetic association, and the difference of HWE between the cases and the controls can be used to test for association^41^. Minor allele frequency (MAF) is the frequency of the less (or least) frequent allele in a given locus and a first reported population. The funnel plot asymmetry was calculated by the Egger’s linear regression test, which is a linear regression methodology to measure the funnel plot asymmetry on the natural logarithm scale of the OR. Student’s t-test (p-value < 0.05 was maintained as a sign of statistically significant publication bias) was used to determine the significance of the intercept^42^. The entire statistical analysis for the present meta-analysis (pooled analysis) was performed with the help of Comprehensive Meta-analysis (CMA) Version 2 software program (Biostat, USA).

## Additional Information

**How to cite this article**: Khan, S. *et al*. *TNF-α* -308 G > A (rs1800629) Polymorphism is Associated with Celiac Disease: A Meta-analysis of 11 Case-Control Studies. *Sci. Rep.* **6**, 32677; doi: 10.1038/srep32677 (2016).

## Figures and Tables

**Figure 1:**
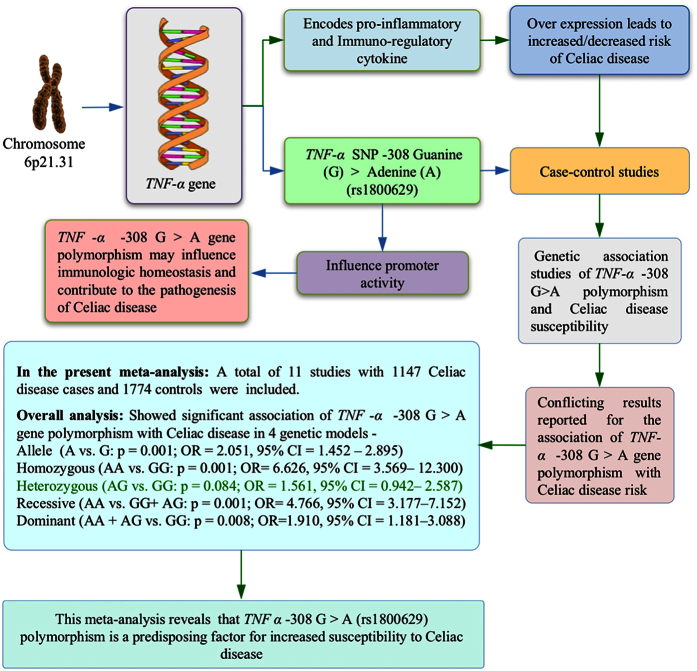
Graphical abstract of the meta-analysis performed to evaluate the association of *TNF-α* -308 G > A (rs1800629) polymorphism and CD susceptibility.

**Table 1:** Summary of major characteristics of all the studies included in the present meta-analysis.

First author and Year of Publication	Country	Ethnicity	Controls	Cases	Study	Technique used	Association Yes/No
Rossi *et al*.^14^	Brazil	Caucasian	267	244	HB	RT-PCR	Yes
de Albuquerque *et al*.^15^	Italy	Caucasian	96	192	HB	PCR-RFLP	Yes
Kekik *et al*.^16^	Turkey	Caucasian	93	33	HB	PCR-SSP	No
Capilla *et al*.^17^	Spain	Caucasian	256	144	HB	GPC	Yes
Hermann *et al*.^18^	Hungary	Caucasian	277	19	HB	PCR-RFLP	No
Barisani *et al*.^19^	Italy	Caucasian	202	155	HB	PCR-RFLP	No
Garrote *et al*.^20^	Spain	Caucasian	99	50	HB	PCR-SSP	No
Lio *et al*.^21^	Italy	Caucasian	220	110	HB	ARMS-PCR	Yes
Cataldo *et al*.^22^	Italy	Caucasian	96	66	HB	PCR Hybridization	Yes
Hahn Zoric *et al*.^23^	Sweden	Caucasian	103	89	HB	PCR-RFLP	No
Garrote *et al*.^24^	Spain	Caucasian	65	45	HB	PCR-RFLP	Yes

HB = Hospital based.

**Table 2:** Genotypic distribution of *TNF-α* -308G > A gene polymorphism included in the present meta-analysis.

Authors and year	Controls				Cases				HWE
	Genotype			Minor allele	Genotype			Minor allele	
	GG	GA	AA	MAF	GG	GA	AA	MAF	p-value
Rossi *et al*.^14^	206	58	3	0.11	107	117	20	0.32	0.62
deAlbuquerque *et al*.^15^	42	46	8	0.32	100	63	29	0.31	0.34
Kekik *et al*.^16^	15	78	0	0.41	16	14	3	0.30	0.01
Capilla *et al*.^17^	191	60	5	0.13	90	44	10	0.22	0.90
Hermann *et al*.^18^	148	118	11	0.25	8	9	2	0.34	0.03
Barisani *et al*.^19^	157	44	1	0.11	85	55	15	0.27	0.25
Garrote *et al*.^20^	72	26	1	0.14	31	18	1	0.2	0.41
Lio *et al*.^21^	163	53	4	0.13	61	34	15	0.29	0.89
Cataldo *et al*.^22^	73	22	1	0.12	44	12	12	0.26	0.64
Hahn zoric *et al*.^23^	70	27	6	0.18	23	44	22	0.49	0.13
Garrote *et al*.^24^	51	14	0	0.10	20	23	2	0.30	0.33

MAF: Minor Allele Frequency; HWE: Hardy Weinberg Equilibrium.

**Table 3:** Quality assessment conducted according to the Newcastle-Ottawa criteria for all the included studies in this meta-analysis.

First author	Year	Quality indicators		
		Selection	Comparability	Outcome
Rossi *et al*.	2015	***	**	**
de Albuquerque *et al*.	2015	****	***	**
Kekik *et al*.	2011	**	*	**
Capilla *et al*.	2007	***	**	***
Hermann *et al*.	2007	***	*	***
Barisani *et al*.	2006	***	**	***
Garrote *et al*.	2005	**	**	***
Lio *et al*.	2005	***	*	***
Cataldo *et al*.	2003	***	*	***
Hahn zoric *et al*.	2003	**	*	***
Garrote *et al*.	2002	****	**	***

**Table 4:** Overall statistics to test publication bias and heterogeneity in the present meta-analysis.

Comparisons	Egger’s regression analysis			Heterogeneity analysis			Model used for the present meta-analysis
	Intercept	95% Confidence Interval	p-value	Q-value	P_heterogeneity_	I^2^ (%)	
A vs. G	−1.96	−8.68 to 4.76	0.52	60.14	0.001	83.37	Random
AA vs. GG	1.16	−1.18 to 3.51	0.29	18.29	0.050	45.35	Fixed
AG vs. GG	−3.37	−9.91 to 3.17	0.27	76.58	0.001	86.94	Random
AA + AG vs. GG	−3.08	−9.82 to 3.66	0.32	76.96	0.001	87.00	Random
AA vs. GG + AG	1.34	−0.37 to 3.07	0.11	12.21	0.271	18.10	Fixed
